# Decoding chemerin proteolytic processing and isoform signaling across disease contexts

**DOI:** 10.1016/j.isci.2026.115259

**Published:** 2026-03-06

**Authors:** Jing Wang, Jiangming Deng, Ting Xiao, Wen Meng

**Affiliations:** 1 Department of Oncology, The Second Xiangya Hospital of Central South University, Changsha, Hunan 410011, China; 2 National Clinical Research Center for Endocrine and Metabolic Diseases and Metabolism and Endocrinology, The Second Xiangya Hospital of Central South University, Changsha, Hunan 410011, China; 3 The Metabolic Syndrome Research Center, Departments of Metabolism and Endocrinology, The Second Xiangya Hospital of Central South University, Changsha, Hunan 410011, China; 4 Department of Hepatopathy and Endocrinology, The Affiliated Children’s Hospital of Xiangya School of Medicine, Central South University (Hunan Children’s Hospital), Changsha, Hunan 410000, China

**Keywords:** health sciences

## Abstract

Chemerin (RARRES2) is a multifunctional adipokine widely implicated in metabolic, inflammatory, cardiovascular, and neoplastic diseases, yet its clinical interpretation remains confounded by reliance on “total chemerin” measurements that obscure its proteoform-specific signaling. This single value is mechanistically misleading because chemerin is secreted as an inactive precursor and undergoes extracellular proteolytic processing into C-terminal isoforms with graded receptor potency and compartment-specific distribution. This review decodes chemerin’s functional duality through three integrated layers: (1) protease-encoded isoform “barcodes” that dictate bioactivity, (2) compartment-specific isoform landscapes in human biofluids and disease microenvironments, and (3) receptor context across CMKLR1, GPR1, and CCRL2 that shapes signaling output. We provide a conceptual roadmap for translating chemerin biology, emphasizing isoform-resolved quantification via targeted /MRM-MS and a compartment-aware framework for interpreting clinical associations. This framework helps interpret heterogeneous disease associations and highlights testable entry points for context-specific targeting.

## Graphical abstract

**Figure:**
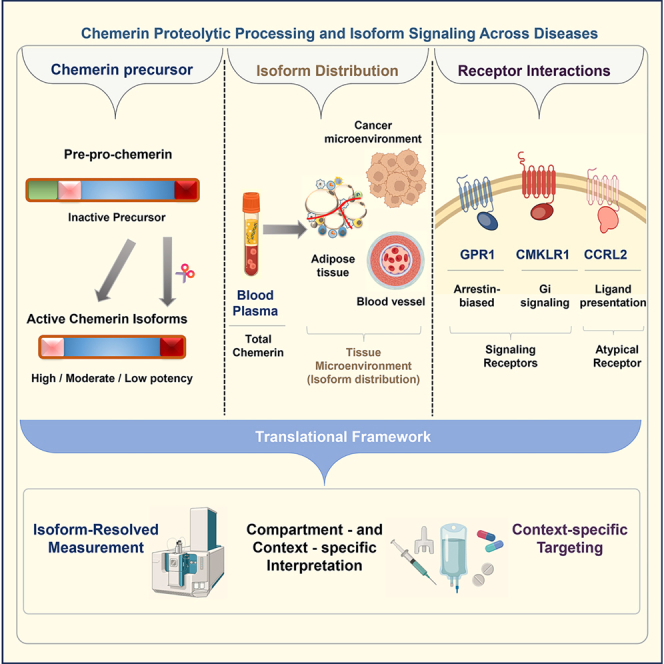

## Introduction

Obesity and related metabolic disorders remain major drivers of morbidity worldwide and contribute to a broad spectrum of complications, including type 2 diabetes, cardiovascular diseases, and obesity-associated cancers.^1^^,^^2^ In these chronic inflammatory and tissue-remodeling states, chemerin is increasingly used as a biomarker candidate; however, associations that vary by compartment (e.g., circulation versus local tissue or biofluids) have limited mechanistic interpretation and translation.^3^^,^^4^ A defining feature is chronic, low-grade inflammation with the endocrine and paracrine reprogramming of adipose tissue, where adipokines coordinate inter-organ crosstalk linking nutrient sensing to immune activation, vascular dysfunction, and tissue injury.^5^

Among adipokines, chemerin has attracted sustained interest because clinical and experimental observations do not fit a simple “higher-is-worse” or “lower-is-better” model. In obesity and metabolic syndrome, circulating chemerin is frequently elevated and associated with metabolic phenotypes,^6^^,^^7^^,^^8^ while weight loss or metabolic interventions can reduce circulating chemerin.^9^^,^^10^^,^^11^ At the same time, chemerin has been reported to exert apparently divergent effects across tissues and disease contexts, including cardiovascular pathology and cancer-related immune regulation.^12^^,^^13^^,^^14^ These inconsistencies have complicated the use of chemerin as a biomarker and have slowed efforts to define when and how chemerin should be targeted therapeutically.

Mechanistically, chemerin biology is shaped by two coupled layers of specificity: proteolytic isoform processing and receptor context. Chemerin was first identified as a retinoid-responsive gene (TIG2/RARRES2)^15^ and later recognized as an adipokine regulating adipogenesis and adipocyte function.^16^ However, chemerin is secreted predominantly as an inactive precursor and requires extracellular C-terminal processing to generate bioactive isoforms.^17^ Proteases involved in coagulation/fibrinolysis and inflammation can sequentially activate or inactivate chemerin, producing isoforms with distinct activities in a tissue- and microenvironment-dependent manner.^18^ Importantly, isoform distributions differ across human compartments; for example, chemerin158K is dominant in synovial and cerebrospinal fluids but not in plasma, supporting the concept that “total chemerin” may not reflect local bioactivity.^19^ Moreover, LC/MRM-MS-based approaches have begun to enable isoform-resolved quantification in human biofluids, highlighting a feasible path toward mechanistically informed clinical measurement.^20^

A second layer of context dependence arises from receptor biology. Chemerin interacts with three receptors—CMKLR1 (ChemR23/ChemerinR1), GPR1 (ChemerinR2), and CCRL2—with distinct signaling properties and biological roles.^21^^,^^22^^,^^23^ CMKLR1 is the canonical Gi/o-coupled signaling receptor, GPR1 is typically arrestin-biased with weak canonical G-protein output, and CCRL2 mainly acts as an atypical binding/presentation receptor that shapes local ligand availability.^21^^,^^24^^,^^25^ While CMKLR1 mediates canonical GPCR signaling, CCRL2 is generally viewed as an atypical receptor that binds chemerin and modulates its local presentation rather than initiating classical signaling.^26^^,^^27^ In contrast, GPR1 generally exhibits *weak/limited* canonical G-protein output in many cellular assays (relative to CMKLR1), while robustly recruiting β-arrestins and undergoing rapid internalization, consistent with roles in arrestin-biased signaling and chemerin scavenging/clearance.^21^^,^^25^^,^^28^^,^^29^

Together, CMKLR1 (classical signaling), GPR1 (arrestin-biased/internalizing), and CCRL2 (presentation) establish a receptor logic that helps explain why identical isoform milieus can yield divergent biological outcomes across tissues and disease microenvironments. The combination of isoform balance, protease milieu, and receptor distribution, therefore, provides a coherent explanation for why chemerin can be associated with heterogeneous or even opposing outcomes across metabolic, inflammatory, cardiovascular, and tumor settings.^30^^,^^31^

**Figure 1:**
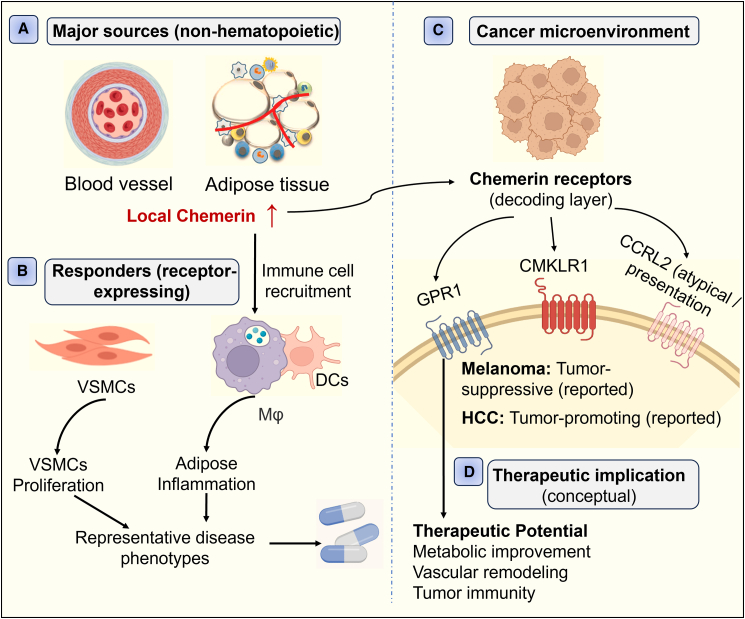
Overview of reported sources and context-dependent actions of chemerin across major disease domains Figure 1 serves as an integrative summary of the isoform-protease-receptor-context framework. Chemerin is produced by adipose and other non-hematopoietic tissue compartments (e.g., stromal/epithelial/endothelial sources reported in specific contexts), whereas leukocytes primarily act as responders via CMKLR1/GPR1 and can shape local chemerin availability through CCRL2. This schematic highlights a conceptual framework and is not intended as a uniform causal model; directionality and outcomes depend on tissue context, receptor expression, and proteolytic isoform composition. Abbreviations: CMKLR1: chemokine-like receptor 1; GPR1: G protein-coupled receptor 1 (ChemerinR2); CCRL2: chemokine C-C motif receptor-like 2; DCs: dendritic cells; Mφ: macrophages; VSMCs: vascular smooth muscle cells; HCC: hepatocellular carcinoma.

In this review, we synthesize current knowledge of chemerin biogenesis, proteolytic activation/inactivation, and receptor-mediated signaling,^8^^,^^26^^,^^32^ and discuss how these mechanisms shape chemerin’s reported roles in metabolic disorders, cardiovascular diseases, chronic inflammatory conditions, and cancer (Figure 1).^1^^,^^2^^,^^13^^,^^14^^,^^31^ Figure 1 provides a conceptual overview of the reported sources of chemerin, its receptor-dependent signaling context, and representative disease-relevant processes across metabolic, cardiovascular, inflammatory, and cancer settings, highlighting that directionality and dominant mechanisms are strongly shaped by compartment and proteolytic processing. We further highlight translational gaps—particularly the limited availability of standardized isoform-resolved measurements and the underuse of receptor/isoform context in human studies^6^^,^^20^—and propose practical directions to improve mechanistic interpretation and therapeutic development.

## Molecular plasticity: The proteolytic barcode encoding functional duality

**Figure 2:**
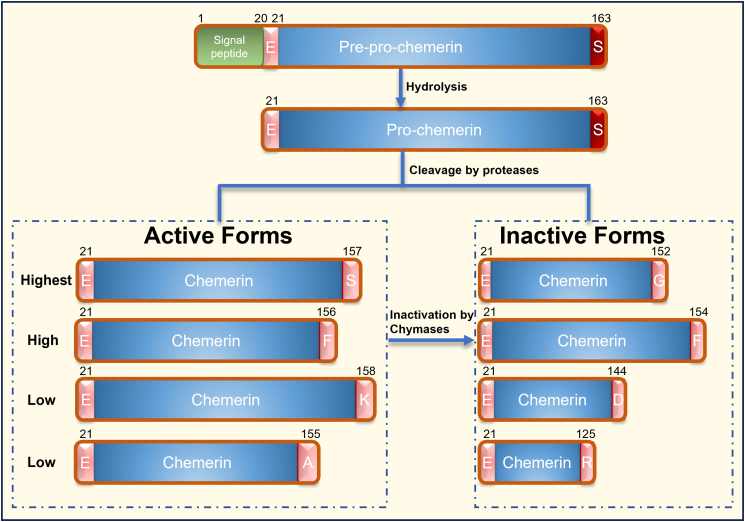
The generation and processing pathway of chemerin and its various isoforms Pre-pro-chemerin (163 aa) is processed by signal peptide cleavage to yield secreted pro-chemerin (Chem-163), which is then C-terminally trimmed by extracellular proteases into isoforms with graded activity. Chem-157 exhibits the highest activity, followed by Chem-156 with slightly lower activity. Chem-155 and Chem-158 possess low activity, while Chem-152, -154, -144, and -125 are relatively inactive. Notably, the active forms of chemerin can undergo further inactivation mediated by chymases.

Chemerin’s frequently “paradoxical” associations across metabolic, vascular, inflammatory, and tumor contexts are best understood as a protease-encoded molecular plasticity rather than intrinsic inconsistency.^33^^,^^34^ Chemerin (RARRES2/TIG2) is synthesized as pre-pro-chemerin (163 aa). Signal peptide cleavage yields the secreted pro-chemerin (Chem-163), which is then C-terminally processed extracellularly into bioactive or inactive isoforms with distinct receptor potency and function.^3^^,^^35^^,^^36^^,^^37^^,^^38^ This principle was established by early work showing that serine proteases from coagulation, fibrinolytic, and inflammatory cascades convert inactive pro-chemerin into bioactive isoforms, thereby coupling chemerin output to the microenvironmental protease milieu rather than to gene expression alone.^17^^,^^37^ Reviews and mechanistic summaries further emphasize that chemerin should be conceptualized as an isoform ensemble whose activity is dynamically regulated by sequential cleavage and trimming events.^8^^,^^32^^,^^33^^,^^36^ Figure 2 provides an overview of stepwise maturation from pre-pro-chemerin (163 aa) to the secreted pro-chemerin (Chem-163) following signal peptide cleavage, and the major C-terminal isoforms generated by distinct protease milieus. The schematic highlights where “activating” versus “terminating” cleavage events occur, offering a mechanistic framework for interpreting why total chemerin levels may not track local bioactivity.

This plasticity is primarily “written” by extracellular proteases from coagulation/fibrinolysis (e.g., plasmin and coagulation/contact proteases), neutrophil proteases (e.g., elastase and cathepsin G), and mast-cell proteases (e.g., tryptase).^17^ Carboxypeptidases further fine-tune C-terminal trimming and can operate in platelet- and plasma-associated settings, while additional proteases such as neutrophil proteinase 3 and mast-cell chymase can also shift chemerin toward less active/inactive forms.^18^^,^^39^ As highlighted in Figure 2, small C-terminal differences create a graded activity spectrum, enabling rapid switching between signaling-competent and inactive chemerin pools depending on the local protease milieu.^38^^,^^40^ A key feature of this “proteolytic barcode” is that small differences at the C-terminus produce large differences in receptor activation. Consistent with this, the C-terminal nonapeptide chemerin-9 (C9) is sufficient to activate CMKLR1, underscoring why proteolytic exposure/trimming of the extreme C-terminus is decisive for signaling potency.^41^ The receptor-specific binding/activation logic (CMKLR1 vs. GPR1 vs. CCRL2) is discussed in Section chemerin and its three receptors: decoding layer. In parallel, neutrophil-driven processing connects chemerin maturation to innate immune activation, providing a mechanistic bridge between acute inflammation and chemerin-dependent leukocyte recruitment.^42^ Importantly, the proteolytic cascade is not unidirectional: Proteases can both activate and inactivate chemerin, creating transient “on” states followed by the termination or redirection of activity. For example, neutrophil proteinase 3 and mast cell chymase participate in chemerin proteolytic regulation, underscoring how inflammatory cell composition can reshape local chemerin bioactivity.^36^^,^^43^^,^^44^ Carboxypeptidase pathways and platelet-dependent regulation add an additional layer by trimming C-terminal residues and modulating the bioactive pool in plasma-associated settings.^39^^,^^45^ Cysteine cathepsins can also regulate chemerin chemoattractant/antibacterial activity, reinforcing the concept that multiple protease families converge on chemerin processing in tissue-specific ways.^46^ Key proteases, cleavage products, and their reported activity spectrum are summarized in Table 1.

**Table 1:** The processing of pro-chemerin

Final Isoform	Precursor Form	Protease	Activity	Reference
Chem-157	Chem-163 (pro-chemerin)	Cathepsin K, Cathepsin L, Elastase	Highest	Zabel; Wittamer; Kulig; Yamaguchi^17^^,^^42^^,^^46^^,^^47^
Chem-156	Chem-163 (pro-chemerin)	Cathepsin G, Chymase	High	Zabel; Guillabert; Wittamer^17^^,^^18^^,^^42^
Chem-158	Chem-163 (pro-chemerin)	Plasmin, Tryptase	Low	Zabel^17^
Chem-155	Chem-163 (pro-chemerin)	Elastase, Proteinase 3	Low	Zabel; Guillabert^17^^,^^18^
Chem-152	Chem-163 (pro-chemerin)	Elastase, Tryptase	Low	Parlee; Du^48^^,^^49^
Chem-154	Chem-157/Chem-156	Chymase	Inactive	Guillabert; Buechler^18^^,^^36^
Chem-125	Chem-163	Cathepsin K, Cathepsin L	Inactive	Zhao; Kulig^32^^,^^46^

Activity classifications are summarized from the cited literature and are typically inferred from receptor-appropriate functional assays, with priority given to Gi/o-mediated cAMP inhibition and chemotaxis for CMKLR1. Calcium mobilization has been reported mainly in heterologous/overexpression systems and should be interpreted cautiously when comparing isoform potency across studies.

Crucially, isoform composition is compartment-specific,^50^^,^^51^ meaning that “total chemerin” in circulation may poorly reflect local functional chemerin signaling.^11^^,^^50^ A pivotal human biofluid study demonstrated that chemerin158K is the dominant chemerin isoform in synovial and cerebrospinal fluids but not in plasma,^19^ providing direct evidence that inflammatory or privileged tissue spaces can harbor isoform landscapes distinct from blood.^50^^,^^52^ In arthritis-related joint fluids, an additional processed form—chemerin156F generated by chymase cleavage—was shown to be elevated, further supporting the idea that inflammatory joint microenvironments contain characteristic chemerin processing signatures.^44^^,^^53^ Together, these observations argue that local protease repertoires (e.g., neutrophil- and mast cell-linked enzymes) can generate functionally divergent tissue-specific chemerin processing signatures, even when total circulating chemerin changes only modestly.^11^^,^^52^

Methodologically, progress in isoform-resolved quantification is beginning to make this framework clinically actionable. An LC/MRM-MS targeted proteomics strategy with stable isotope-labeled standards has been used to distinguish chemerin isoforms in human biofluids, offering a feasible route toward routine profiling of the bioactive versus inactive pool in translational cohorts.^20^ Taken together, the current evidence supports a working model in which chemerin’s functional duality is encoded by protease context, executed through isoform-specific receptor engagement, and observable only when measurements move beyond bulk “total chemerin” toward isoform-aware profiling.^17^^,^^20^^,^^32^^,^^36^^,^^51^^,^^52^

Beyond proteolytic processing, chemerin biology is also regulated at the transcriptional level through RARRES2 gene expression control, and at the protein clearance level through receptor-mediated internalization and degradation.^16^^,^^30^^,^^32^ These additional regulatory layers, while not the focus of this review, contribute to overall chemerin homeostasis and may interact with protease-mediated regulation in complex ways.

In addition to its roles in metabolic and inflammatory regulation, chemerin and its proteolytic fragments contribute to antimicrobial defense. Chemerin-derived peptides exhibit direct antimicrobial activity against bacteria and fungi, and chemerin processing by cysteine cathepsins has been linked to its antibacterial functions.^54^^,^^55^^,^^56^ This antimicrobial dimension further underscores the functional diversity generated by proteolytic processing.

## Chemerin and its three receptors: Decoding layer

Chemerin signals through three distinct receptors with differential signaling properties, tissue distribution, and biological outputs: CMKLR1 (ChemR23/ChemerinR1), GPR1 (ChemerinR2), and CCRL2.

CMKLR1 (ChemR23/ChemerinR1) is the best-characterized chemerin receptor and functions as a classical G protein-coupled receptor. Upon chemerin binding, CMKLR1 couples primarily to Gi/o proteins, leading to the inhibition of adenylate cyclase and reduction of intracellular cAMP levels.^24^^,^^29^ Calcium mobilization and ERK/MAPK activation have been observed in multiple assay formats (including heterologous expression systems and some primary leukocytes); however, across systems, the most consistent hallmark of Gi/o coupling is the inhibition of forskolin-stimulated cAMP.^21^^,^^57^ CMKLR1 is widely expressed in adipose tissue, immune cells (including dendritic cells, macrophages, and NK cells), vascular endothelial cells, and certain neuronal populations.^16^^,^^58^^,^^59^^,^^60^ Through these signaling pathways, CMKLR1 mediates chemerin’s effects on adipocyte differentiation, leukocyte chemotaxis, and inflammatory responses.^29^

GPR1 (ChemerinR2) displays distinct signaling properties compared to CMKLR1. Recent structural work resolved GPR1 bound to full-length chemerin and revealed a chemokine-like reverse binding mode, providing a structural framework for interpreting receptor selectivity and signaling bias.^61^ While GPR1 can weakly activate G protein pathways, its predominant response to chemerin involves β-arrestin recruitment and rapid receptor internalization. This arrestin-biased signaling profile positions GPR1 as a potential chemerin scavenger or clearance receptor in certain contexts, although arrestin-mediated signaling can also activate distinct downstream pathways, including ERK and Akt. GPR1 is expressed in metabolically relevant tissues, including adipose, liver, and skeletal muscle, as well as in reproductive tissues and certain brain regions. Importantly, GPR1’s lack of robust canonical G protein signaling distinguishes it functionally from CMKLR1, and this distinction has implications for interpreting tissue-specific chemerin responses.^21^^,^^25^^,^^28^^,^^29^

CCRL2 functions as an atypical chemerin receptor that does not initiate classical G protein signaling.^21^^,^^62^ Instead, CCRL2 binds chemerin efficiently and is thought to modulate local chemerin availability by presenting the ligand to signaling-competent receptors or by concentrating chemerin in specific tissue microenvironments.^63^ CCRL2 is expressed on endothelial cells, certain epithelial populations, and subsets of leukocytes.^27^ In the vascular endothelium, CCRL2 expression is upregulated by pro-inflammatory stimuli through nuclear factor kappa B (NF-κB) and JAK/signal transducers and activators of transcription (STAT) pathways, suggesting a role in regulating chemerin bioavailability during inflammatory responses.^64^

Together, these three receptors create a layered signaling system in which tissue-specific receptor expression determines whether chemerin exposure results in classical GPCR signaling (CMKLR1), arrestin-biased responses (GPR1), or modulation of local ligand availability (CCRL2). This receptor heterogeneity provides a mechanistic basis for context-dependent chemerin effects across tissues and disease states.

## Why total chemerin fails as a biomarker: the need for isoform-resolved interpretation

Standard immunoassays typically report total chemerin without resolving the relative abundance of inactive precursor versus bioactive C-terminal isoforms, collapsing mechanistic heterogeneity into a single composite number.^4^^,^^52^ This technical limitation is increasingly solvable with targeted proteomics approaches capable of isoform discrimination.^20^

In metabolic disease, multiple studies indicate that chemerin correlates with obesity-linked phenotypes and metabolic dysfunction, and chemerin can decrease after substantial weight loss. For example, chemerin correlates with fatty liver markers in morbid obesity and strongly decreases after bariatric surgery-induced weight loss,^9^^,^^65^ while chemerin is associated with metabolic syndrome phenotypes in population studies.^66^^,^^67^ Chemerin has also been discussed as a mediator linking obesity to vascular inflammation in pediatric settings, supporting its broader relevance to cardiometabolic risk biology.^68^ However, without isoform resolution, these clinical signals cannot distinguish whether elevated total chemerin reflects (1) increased production of largely inactive precursor, (2) a protease milieu favoring bioactive forms, or (3) shifts toward compartment-specific isoforms that may never be captured by plasma measurements.^32^^,^^36^ Recent clinical analyses in participants with and without insulin resistance/diabetes continue to expand the observational base, but they also reinforce the need to interpret associations through an isoform-aware lens rather than assuming that total chemerin equates to uniform receptor activation.^6^

The same interpretive problem becomes more acute when chemerin is considered for prognosis or therapy across divergent diseases, where the direction of effect can plausibly differ by tissue and cellular receptor context. In chronic kidney disease and vascular pathology, chemerin has been reported to inhibit vascular calcification through ChemR23/CMKLR1 and to associate with lower coronary calcium, suggesting a protective axis in specific vascular contexts.^12^ In hepatocellular carcinoma, chemerin has been described as protective through mechanisms that include limiting inflammatory cytokine programs and suppressing MDSC accumulation,^13^ and through a CMKLR1-PTEN-Akt axis that restrains metastasis.^14^ These examples do not imply that chemerin is uniformly protective or harmful; rather, they illustrate that disease outcome depends on which chemerin forms are present, which proteases dominate, and where signaling-competent receptors are expressed—variables that are invisible to total-chemerin assays.

**Figure 3:**
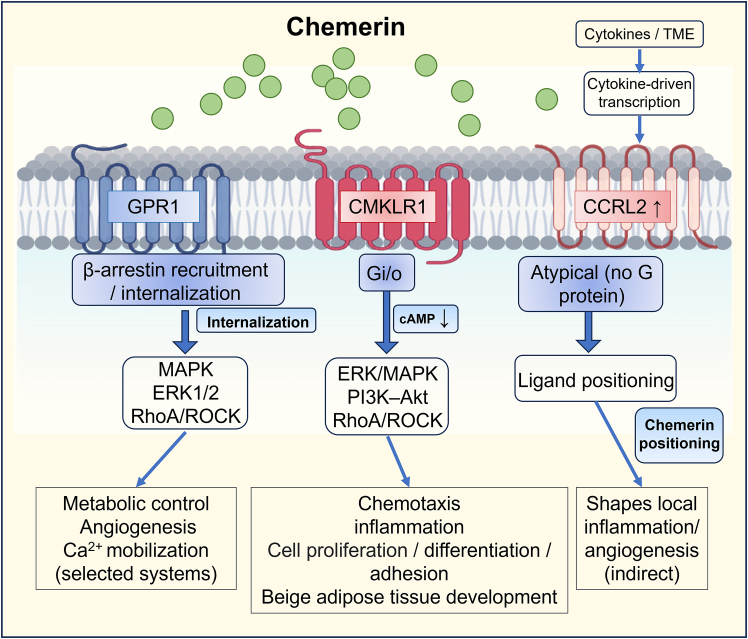
Representative receptor-associated signaling and reported biological outputs of chemerin receptors Upon binding to CMKLR1, chemerin triggers Gi/o-coupled signaling, leading to the inhibition of adenylate cyclase (reduced cAMP), and can activate downstream pathways, including MAPK/ERK and PI3K-Akt. In artificial overexpression systems, CMKLR1 activation has been associated with calcium mobilization, although Gi/o-coupled signaling primarily regulates cAMP in native systems. GPR1 preferentially recruits β-arrestin upon chemerin binding, leading to receptor internalization and arrestin-mediated signaling outputs, including ERK activation. CCRL2 binds chemerin but does not trigger canonical G-protein signaling; it mainly presents chemerin to neighboring CMKLR1/GPR1-expressing cells. In endothelial cells, CCRL2 expression is induced by NF-κB and JAK/STAT signaling downstream of pro-inflammatory stimuli, supporting its role in vascular ligand presentation. Abbreviations: CMKLR1: chemokine-like receptor 1 (ChemR23/ChemerinR1); GPR1: G protein-coupled receptor 1 (ChemerinR2); CCRL2: C-C chemokine receptor-like 2; MAPK: mitogen-activated protein kinase; JAK/STAT: Janus kinase/signal transducers and activators of transcription; NF-κB: nuclear factor-κB.

This dichotomy is further amplified by receptor context: CMKLR1 and GPR1 are signaling-competent GPCRs, whereas CCRL2 primarily functions as an atypical binding/presentation receptor that shapes local ligand availability. Importantly, receptor engagement is isoform-sensitive: Recent structural and mutational analyses have defined activation determinants for CMKLR1, indicating that small changes at the chemerin C-terminus can shift receptor-contact geometry and signaling efficacy.^24^^,^^69^ Chem-155 (chem155A) has been reported to behave as a weak antagonist or low-efficacy agonist at CMKLR1 in specific assay settings, underscoring that closely related isoforms are not functionally interchangeable.^47^ Consistently, the short C-terminal peptide ligand C9 can still activate/modulate chemerin-receptor responses, reinforcing C-terminal cleavage as a functional hotspot—and explaining why total chemerin cannot predict signaling-competent ligand pools.^41^ In activated vascular endothelium, CCRL2 is co-upregulated with vascular cell adhesion molecule 1 (VCAM-1) under pro-inflammatory stimulation, and CCRL2 induction requires NF-κB and Janus kinase/STAT signaling, supporting a role for endothelial CCRL2 in local chemerin presentation and leukocyte-endothelial interactions.^64^ These receptor-associated pathways and representative downstream outputs are summarized in Figure 3.

To anchor the schematic in primary evidence, two examples illustrate how receptor context shapes downstream outputs. In endothelial cells, pro-inflammatory stimuli co-induce CCRL2 and VCAM-1, and CCRL2 induction is dependent on NF-κB and JAK/STAT signaling, consistent with a role for CCRL2 in vascular ligand presentation and leukocyte-endothelial interactions.^64^ In metabolic tissues, CMKLR1 can sense chemerin and the specialized pro-resolving mediator resolvin E1, and experimental studies have linked CMKLR1 signaling to the regulation of adipose thermogenic programs with mTORC1 as a mechanistic node in these models.^70^

Accordingly, “clinical chemerin myopia” is best viewed as a solvable translational gap: Total-chemerin associations should be complemented by isoform-resolved measurements interpreted in the relevant compartment. Practically, this means pairing precursor-versus-processed isoform profiling (e.g., LC/MRM-MS or isoform-selective immunoassays) with receptor/protease context to infer signaling-competent chemerin pools.

## Therapeutic opportunities: Tissue-, receptor-, and isoform-aware targeting of the chemerin axis

To transcend “chemerin myopia,” therapeutic thinking must move beyond globally increasing or decreasing total chemerin and instead adopt a context-specific strategy anchored in chemerin’s mechanistic grammar. As summarized in Figure 2 and Table 1, chemerin is secreted largely as an inactive precursor and becomes biologically meaningful only after microenvironment-dependent proteolytic editing into isoforms with graded activity. In parallel, Figure 3 highlights that chemerin biology is further diversified by receptor context: CMKLR1 and GPR1 can transduce signaling, whereas CCRL2 primarily shapes ligand availability and spatial presentation. Taken together, these considerations argue that the most credible translational objective is not “chemerin up” or “chemerin down,” but isoform- and receptor-aware modulation in the relevant compartment.

### Tissue-targeted CMKLR1 modulation for metabolic benefit

Chemerin was initially positioned as an adipokine that regulates adipogenesis and adipocyte metabolism, providing a mechanistic rationale for its association with obesity-related phenotypes.^16^ In human cohorts, circulating chemerin is often elevated in obesity/metabolic syndrome and can decline following weight loss or combined lifestyle interventions, consistent with a state-dependent regulation of the chemerin axis.^11^^,^^65^^,^^66^ However, these observations do not imply that systemic CMKLR1 activation is automatically beneficial. Beyond its inflammatory roles, CMKLR1 signaling has also been implicated in adipose energy homeostasis; for example, CMKLR1 sensing of chemerin/resolvin E1 has been linked to the modulation of adipose thermogenesis, with mTORC1-related signaling proposed as one contributing pathway in experimental models.^70^ CMKLR1 is expressed beyond adipocytes, and chemerin-CMKLR1 signaling has been implicated in inflammatory and vascular settings, including diabetic cardiomyopathy-associated inflammatory programs and atherosclerosis-related immune phenotypes.^71^^,^^72^^,^^73^ Thus, the key translational challenge is compartmental specificity: Metabolic benefit is most plausibly achieved when CMKLR1 modulation is preferentially restricted to metabolic tissues, while minimizing unintended activation in immune/vascular compartments.

A practical implication is that future CMKLR1-directed pharmacology should be evaluated with explicit safety-relevant readouts that capture immune and vascular activation, rather than relying solely on metabolic endpoints. Furthermore, as ligand potency depends on isoform composition, pharmacodynamic interpretation must incorporate isoform-aware measurement (Section translational toolkit: isoform-resolved assays, receptor readouts, and compartment-aware study design) to avoid conflating changes in total chemerin with changes in signaling-competent chemerin pools.

Beyond metabolic applications, CMKLR1 targeting is being explored for cancer diagnostics and therapy. For example, CMKLR1 has been evaluated as an imaging target, and CMKLR1-focused pharmacology is being developed across tumor contexts. Although selectivity and on-target validation remain critical for interpretation,^74^^,^^75^^,^^76^ and recent structure-/screening-guided efforts have also yielded small-molecule antagonists with defined binding modes for CMKLR1 (and related insights for GPR1).^77^ Because ligand potency is isoform-dependent, we discuss isoform-receptor coupling and why C-terminal processing matters in Section chemerin and its three receptors: decoding layer.

### Chemerin receptors in cancer: from ligand presentation to signaling outputs

Chemerin’s associations in cancer are heterogeneous across tumor types and clinical contexts, consistent with the idea that chemerin output depends on (1) receptor context and (2) the immune architecture and proteolytic milieu of the tumor microenvironment.^78^ For example, chemerin expression has been linked to prognosis in breast cancer^79^ and has been discussed as a biomarker node at the interface of inflammation, chemotaxis, and coagulation/fibrinolysis in resectable non-small cell lung cancer.^80^

Within this framework, CCRL2 deserves particular attention: It binds chemerin but is generally considered an atypical receptor that modulates local ligand availability rather than inducing canonical GPCR signaling.^27^^,^^63^ Specialized lung capillary endothelial CCRL2 has been shown to control NK-cell homing in lung cancer,^81^ supporting a model in which CCRL2 shapes immune-cell access to tumors by regulating chemerin availability in the tumor vasculature. Complementing this, chemerin can trigger the migration of a CD8 T cell subset with natural killer-like functions,^82^ providing a mechanistic basis for how local chemerin presentation can influence antitumor effector recruitment in selected contexts.

However, tumor outcomes are unlikely to be dictated by CCRL2 alone. In immune-permissive settings, CMKLR1 expressed on infiltrating leukocytes can function as the signaling receptor that translates local chemerin availability into chemotaxis and effector programs, whereas GPR1, with its arrestin-biased/internalizing profile, has been proposed as an actionable tumor-associated node in selected contexts (e.g., receptor-selective engagement beyond CCRL2-mediated presentation).^24^^,^^83^^,^^84^

Translationally, these points motivate a more precise hypothesis than “increase chemerin in cancer.” Instead, strategies can be framed around where chemerin is presented (CCRL2-dependent ligand handling) and which signaling nodes are engaged (CMKLR1/GPR1), and should be paired with isoform- and receptor-resolved pharmacodynamic readouts to verify that signaling-competent chemerin is actually available to the intended effector cells.

### Reprogramming the proteolytic milieu: Isoform steering as a therapeutic logic

Unlike many cytokines, chemerin is heavily regulated at the post-secretory level by proteolytic cascades, including coagulation/fibrinolysis proteases and inflammatory proteases, which can both activate and terminate chemerin bioactivity.^18^^,^^39^^,^^42^ Additional protease families, such as cysteine cathepsins, further expand tissue-specific routes for shaping chemerin’s chemoattractant and antimicrobial activity.^46^ This biology implies that, in principle, the “druggable intermediate” may not be chemerin abundance per se, but the balance between signaling-competent and inactive isoforms created in a given lesion.

In vascular disease, for example, chemerin has been reported to inhibit vascular calcification through ChemR23/CMKLR1 and to associate with lower coronary calcium in chronic kidney disease, suggesting that chemerin signaling can be protective in specific vascular contexts.^12^ However, the precise *in vivo* isoforms mediating such effects remain incompletely resolved, underscoring why therapeutic proposals must avoid assuming that one isoform (e.g., Chem-157) is uniformly “pathogenic” or “beneficial” across tissues. A more defensible translational direction is therefore isoform steering: defining which protease activities dominate within a target compartment and determining whether shifting the local processing balance (toward or away from specific isoforms) aligns with the desired outcome. This is ultimately an empirical question that depends on compartment-specific isoform profiling and protease context mapping (Section translational toolkit: isoform-resolved assays, receptor readouts, and compartment-aware study design).

### From mechanistic insight to testable therapeutic hypotheses

While direct clinical validation remains limited, the following conceptual framework proposes testable hypotheses for context-specific intervention. The strategies outlined above can be unified into a practical “context matrix” requiring three elements: (1) isoform-resolved quantification, (2) receptor mapping in relevant compartments, and (3) protease-context profiling. This framework follows directly from the mechanistic considerations established in Figures 2 and 3 and Table 1, and is further detailed in Section translational toolkit: isoform-resolved assays, receptor readouts, and compartment-aware study design.

## Translational toolkit: Isoform-resolved assays, receptor readouts, and compartment-aware study design

The major translational barrier in chemerin research is not the absence of associations, but the absence of mechanistically interpretable measurements. Conventional immunoassays typically capture “total chemerin” and cannot distinguish inactive precursor from processed bioactive isoforms. Therefore, a credible translational toolkit must be built around isoform resolution, receptor-aware functional readouts, and compartment-anchored sampling.

First, isoform-resolved quantification is essential. Multiple analytical approaches can distinguish chemerin isoforms: Targeted LC/MRM-MS with stable isotope-labeled standards provides direct isoform identification and quantification in human biofluids^20^; immunoaffinity-based methods with C-terminal-specific antibodies can distinguish between pro-chemerin and processed forms; and top-down proteomics approaches enable comprehensive characterization of the chemerin proteoform landscape. Each method has distinct advantages in sensitivity, throughput, and quantitative accuracy, and method selection should be guided by study scale and available infrastructure.

Second, receptor-anchored functional assays should match the native signaling context. For CMKLR1, physiologically relevant assays include Gi/o-mediated cAMP inhibition, chemotaxis, and downstream kinase activation measured in cells expressing endogenous receptor levels. While calcium flux has been reported in artificial overexpression systems, CMKLR1’s primary signaling output in native contexts is Gi/o-mediated cAMP regulation. For GPR1, β-arrestin recruitment and receptor internalization assays are more appropriate given its arrestin-biased signaling profile. For CCRL2, functional readouts should focus on ligand binding and presentation rather than canonical signaling outputs. Critically, assays should use chemerin isoforms isolated from the tissue or disease compartment under study, rather than recombinant forms alone, to capture native activity profiles.

Third, protease context mapping should be incorporated. Understanding which serine proteases are present in a given tissue (e.g., neutrophil elastase, mast cell chymase, and plasmin) is essential for interpreting chemerin isoform activity. Methods such as protease activity assays, zymography, and protease-specific activity-based probes can help define the local proteolytic environment.

Fourth, receptor expression profiling is needed. Methods including qPCR, western blotting, flow cytometry, and immunohistochemistry should be used to determine which chemerin receptors (CMKLR1, GPR1, and CCRL2) are expressed in the tissue or cell population under study, as receptor context determines which signaling pathways can be engaged.

Taken together, these components can help move the field from correlative “total chemerin” associations toward testable, context-defined hypotheses, provided that isoform and receptor readouts are implemented in the relevant compartments.

## Conclusions and outlook: From observation to mechanistic decoding

### Conclusion

Chemerin’s apparent paradox across diseases should be viewed less as a contradiction and more as a consequence of context-dependent signal encoding and decoding. Figure 2 and Table 1 illustrate how protease-dependent processing generates an isoform landscape with graded activity, while Figure 3 shows that receptor context determines which biological programs are ultimately engaged. This framework explains why total circulating chemerin can yield inconsistent clinical associations: bulk assays collapse inactive precursor and processed bioactive isoforms into a single readout and ignore compartment-specific isoform dominance demonstrated in human biofluids.

A pragmatic path forward, therefore, follows directly from existing evidence. The field should prioritize isoform-resolved profiling in relevant compartments, paired with receptor-anchored functional readouts that distinguish signaling from ligand handling. Clinical studies should interpret state-dependent changes in chemerin—such as declines observed after weight loss and lifestyle interventions—through this isoform- and receptor-aware lens rather than assuming that total chemerin represents uniform receptor activation.^65^^,^^85^ Finally, therapeutic proposals should be framed as testable, compartment-specific hypotheses that align with the context matrix: CMKLR1 modulation evaluated with immune/vascular safety awareness, CCRL2-focused strategies justified by evidence for vascular gatekeeping of immune trafficking in cancer, and protease-context approaches grounded in direct isoform and protease profiling rather than assumed isoform effects.

In summary, chemerin’s dualities are not a barrier to translation; they are an invitation to measure and intervene with higher resolution. Once isoform composition, protease context, and receptor landscape are treated as core variables rather than peripheral details, chemerin can be repositioned from a paradoxical biomarker to a stratifiable pathway with rational, context-dependent therapeutic entry points. An integrative summary schematic that links isoform generation, protease context, receptor decoding, and compartment-dependent outputs is provided in Figure 1.

### Major unanswered questions and future directions

Several key questions remain in chemerin biology: (1) What is the predominant chemerin isoform in humans under various physiological and pathological conditions? (2) How safe and effective would targeted protease modulation be for controlling chemerin activity? (3) What are the clinical translation barriers for isoform-resolved measurements? (4) How can we develop therapeutic strategies that account for compartment-specific isoform distributions? Addressing these questions will require coordinated efforts in analytical method development, clinical cohort studies, and translational research.

## Acknowledgments

This work was partially supported by grant 82370858 and 82170886 from the 10.13039/501100001809National Natural Science Foundation of China, 2023JJ10090 from the Distinguished Young Scholar Foundation of Hunan Province, 2025JJ50705 from the 10.13039/501100004735Natural Science Foundation of Hunan Province.

## Author contributions

J.W. drafted the manuscript. W.M. conceptualized and revised the manuscript. J.D. and T.X. reviewed and edited the manuscript. All authors approved the final version.

## Declaration of interests

The authors declare no competing interests.
